# Supplementary material for: Inactivation of MexT in Pseudomonas aeruginosa PAO1 destabilizes cooperation and favors the emergence of a unique quorum sensing variant
Source: J Bacteriol. 2026 Apr 22;208(5):e00434-25. doi: 10.1128/jb.00434-25 (PMC13182392; doi:10.1128/jb.00434-25)
Supplement: Supplemental figures — Figures S1 to S3. [file jb.00434-25-s0001.pdf]

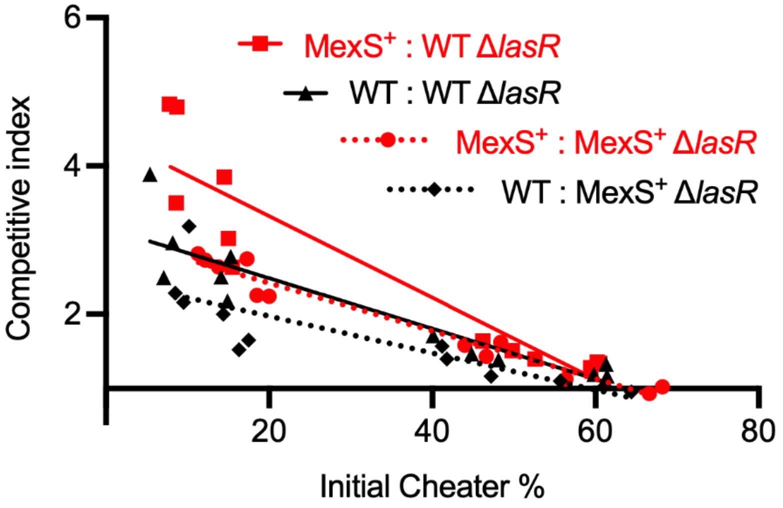

Figure S1: Competitive indices from coculture competitions with varying starting frequencies. WT (black) or $MexS^+$ (red) cooperators were competed against LasR-null mutants without (solid lines) or with (dotted lines) the $MexS^+$ allele. Competitions started with the initial concentration of cheaters at approximately 10%, 15%, 45%, and 60% of the population, and the data points represent the competitive indices at 24 h calculated from the actual initial frequency.

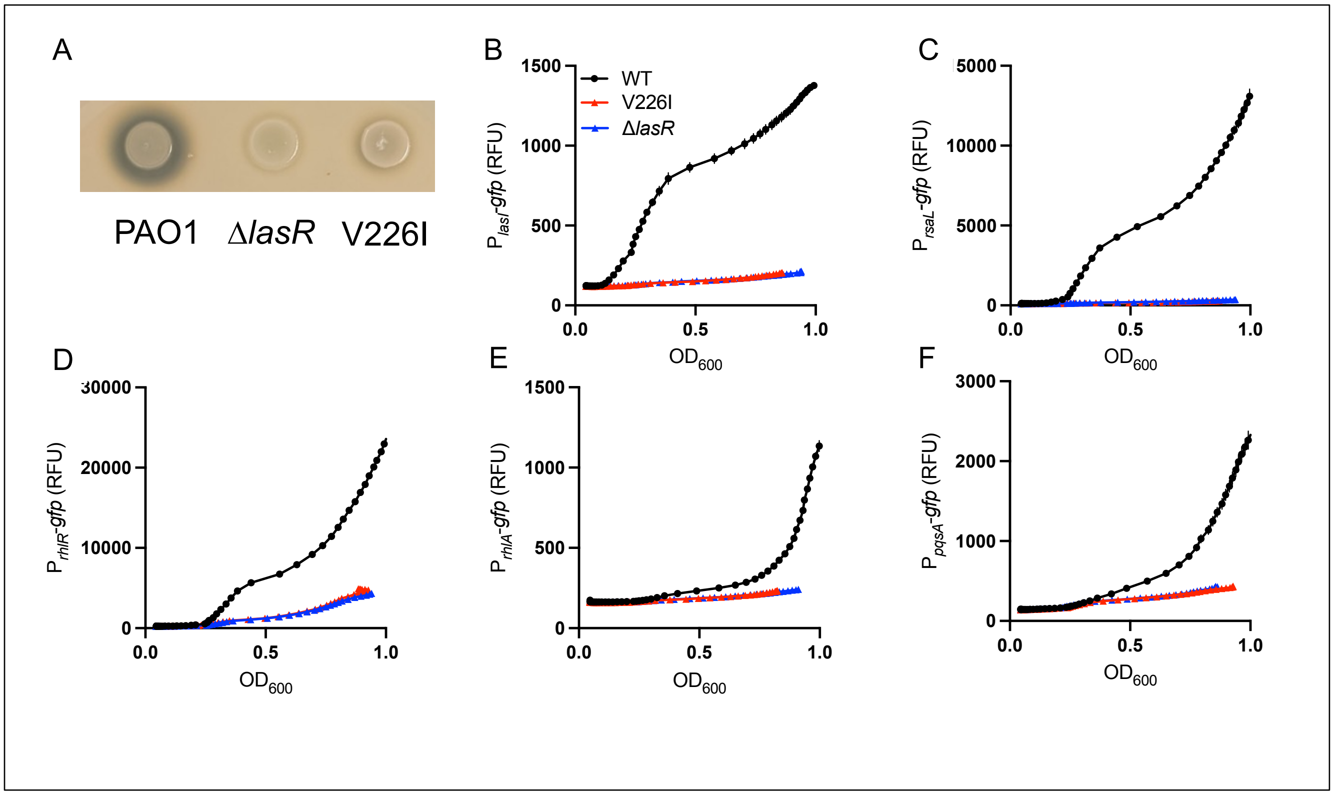

Figure S2. WT, LasR-V226I, and Δ*lasR* strain phenotypes in pure cultures. **A**) Colonies on skim milk agar. The zones of clearance represent protease production. **B-F**) Time course assays of gene expression in LB: *lasI* (**B**), *rsaL* (**C**), *rhlR* (**D**), *rhlA* (**E**), and *pqsA* (**F**). Data are means of four replicates, and the error bars represent SD.

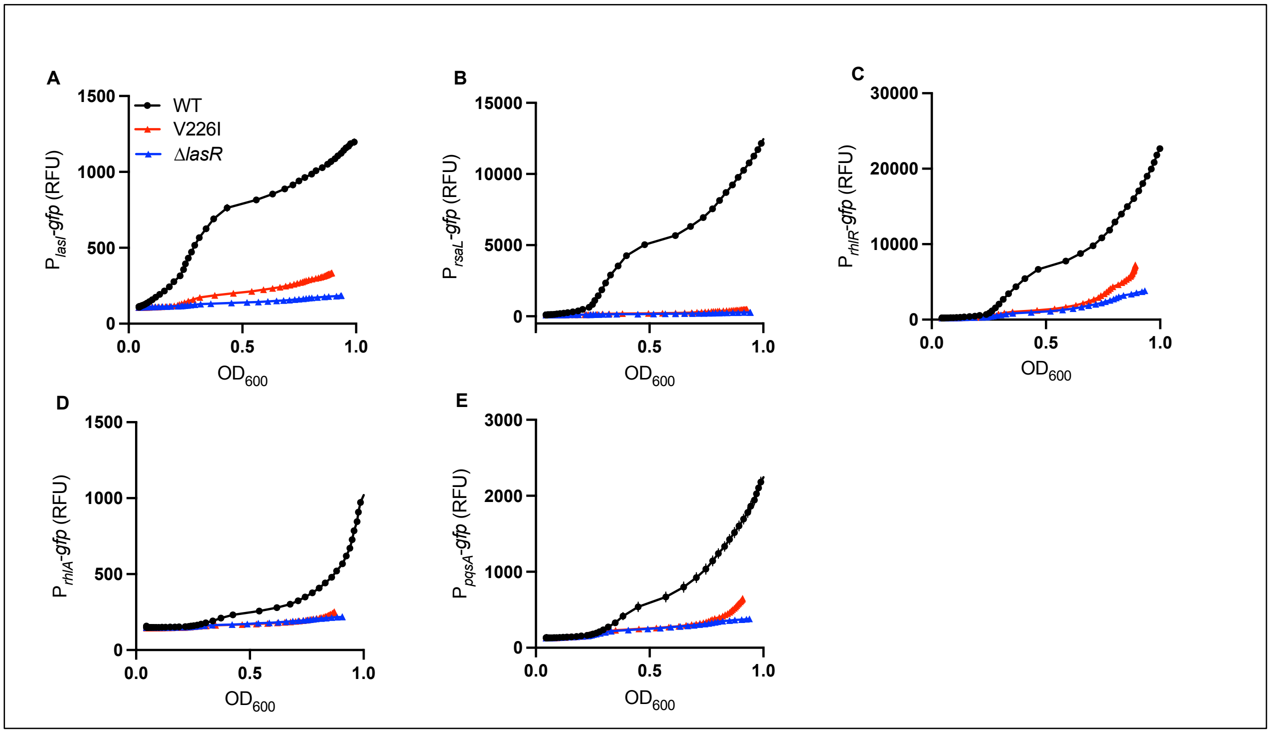

Figure S3. Time course assays of gene expression in LB supplemented with 5 µM 3OC12-HSL: *lasI* (**A**), *rsaL* (**B**), *rhlR* (**C**), *rhlA* (**D**), and *pqsA* (**E**). Data are means of four replicates. Error bars representing SD are too small to see.
