# Supplementary material for: Inactivation of MexT in Pseudomonas aeruginosa PAO1 destabilizes cooperation and favors the emergence of a unique quorum sensing variant
Source: J Bacteriol. 2026 Apr 22;208(5):e00434-25. doi: 10.1128/jb.00434-25 (PMC13182392; doi:10.1128/jb.00434-25)
Supplement: Supplemental tables — Tables S1 to S3. [file jb.00434-25-s0002.pdf]

Table S1. Whole genome sequencing of isolates from each background to identify mutations in genes other than *lasR.*

Background[a]	Day of collection	Protease production[b]	LasR AA sequence[c]	Other mutations[d]	DNA change[e]	AA change[f]
WT; 2C	28	Negative	Wildtype	*gacA*	C350T	Q117*[g]
				psdR	C31T	R11C
				pilB	T665A	L222H
				rpoA	A902G	E301G
WT; 2B	28	Negative	V226I	*psdR*	G73C	A25P
$MexS^+$; 2B	30	Positive	V226I	*gacA*	Δ13 bp	Frameshift, V7D
				psdR	A490G	N164D
				pilQ	C1990T	Q664*
$MexS^+$; 2B	20	Negative	F210L	*fleQ*	Δ12bp	Frameshift, G226S
$MexT^—$; 2A	30	Positive	V226I	*gacS*	Δ1 bp	Frameshift, N267T
				psdR	A479T	H160L
				pilQ	G1161A	Q329*
				pvcC	C854T	A285V
$MexT^—$; 2B	30	Negative	L160*	*fleQ*	C527T	S176F
				psdR	G398A	G133E

[a]Background of isolates obtained from the second of two 30-day passaging experiments from the A, B, or C biological replicate.
[b]Protease production as determined by patching single colonies onto skim milk and incubating overnight at 37˚C. Positive colonies generated a zone of clearing around the colony, indicating digestion of milk protein by proteases, while negative colonies did not.
[c]Protein sequence change from wildtype PAO1 LasR (1).
[d]Single nucleotide polymorphisms (SNPs) and insertion/deletion mutations identified by *breseq* variant analysis (2) with a read frequency of 95% or higher using Stover PAO1 as the reference. SNPs called in the initial analysis were further compared to the UW parent PAO1 reference sequence to identify mutations present in the lab strain before starting the passaging experiment.
[e]For genes with mutations, loci of nucleotide changes with respect to CDS start as identified at pseudomonas.com (3).
[f]Amino acid change resulting from mutation in protein coding sequence.
[g]Asterisks denote an amino acid change resulting in an early stop codon at the referenced residue.

Table S2. Plasmids used in this study.

Plasmid	Description	Source
pBBR1MCS-5	Broad-host-range expression plasmid, Gm^R	(4)
pBBR-MCS-*gfp*	Promoter-less *gfp* transcriptional reporter, Gm^R	(5)
pP$_{lasI}$-*gfp*	pBBR1MCS-5 with *lasI* promoter fused to *gfp*, Gm^R; encodes -282 to +223 relative to the start of *lasI* and includes the complete *rsaL* binding site	(6)
pP$_{pqsA}$-*gfp*	pBBR1MCS-5 with *pqsA* promoter fused to *gfp*, Gm^R; encodes -429 to +3 relative to the start of *pqsA*	(5)
pP$_{rhlA}$-*gfp*	pBBR1MCS-5 with *rhlA* promoter fused to *gfp*, Gm^R; encodes -500 to +31 relative to the start of *rhlA*	(6)
pP$_{lasB}$-*gfp*	pBBR1MCS-5 with *lasB* promoter fused to *gfp*, Gm^R	(6)
pUC18T-mini-Tn7T-Gm-*gfp*	Mini-Tn7-based vector for chromosomal integration of *gfp* and gentamicin resistance cassette at neutral *att* site; Gm^R	(7)
pTNS2	Helper plasmid for mini-Tn7-based chromosomal integration	(7)
pUC18T-mini-Tn7T-Gm-mCherry	Mini-Tn7-based vector for chromosomal integration of mCherry and gentamicin resistance cassette at neutral *att* site; Gm^R	(8)
pEXG2-Δ*lasR*	pEXG2 allelic exchange vector with sequence for 711 bp deletion of *lasR*; preserves last 6 bp of *rsaL*; *sacB,* Gm^R	(9)
pEXG2-LasR-V226I	pEXG2 allelic exchange vector to introduce the G676A mutation in *lasR*; *sacB,* Gm^R	This work

Gm^R ; Gentamicin resistance

Table S3. Strains used in this study.

Strain	Description	Source
P. aeruginosa		
PAO1	Wild type laboratory strain with A745G mutation in *mexS* resulting in constitutive MexT activity	(1)
PAO1 Δ*lasR*	PAO1 derivative with unmarked, in-frame *lasR* deletion	(9)
PAO1 MexT⁻	PAO1 derivative with unmarked, in-frame *mexT* deletion	(6)
PAO1 $MexS^+$	PAO1 derivative with restored *mexS* (A745)	(6)
PAO1 $MexS^+$ Δ*lasR*	PAO1 $MexS^+$ with unmarked, in-frame *lasR* deletion	This work
PAO1 LasR-V226I	PAO1 derivative with partially functional LasR variant V226I	This work
PAO1 ΔlasR-*gfp*	PAO1 Δ*lasR* with chromosomally integrated, constitutively expressed *gfp*	(10)
PAO1 Δ*lasR* mCherry	PAO1 Δ*lasR* with chromosomally integrated, constitutively expressed mCherry	(11)
PAO1 $MexS^+$ Δ*lasR*-*gfp*	PAO1 $MexS^+$ Δ*lasR* with chromosomally integrated, constitutively expressed *gfp*	This work
PAO1 LasR-V226I-mCherry	PAO1 LasR-V226I with chromosomally integrated, constitutively expressed mCherry	
E. coli		
NEB5α	*fhuA2 Δ(argF-lacZ)U169 phoA glnV44 Φ80 Δ(lacZ)M15 gyrA96 recA1 relA1 endA1 thi-1 hsdR17*	NEB
S17-1	*recA* pro *hsdR* RP4-2Tc::Mu-Km::Tn7	(12)

Supplemental Refences

1. Jacobs MA, Alwood A, Thaipisuttikul I, Spencer D, Haugen E, Ernst S, Will O, Kaul R, Raymond C, Levy R, Chun-Rong L, Guenthner D, Bovee D, Olson MV, Manoil C. 2003. Comprehensive transposon mutant library of *Pseudomonas aeruginosa*. Proc Natl Acad Sci 100(24):14339-44.

2. Deatherage DE, Barrick JE. 2014. Identification of mutations in laboratory-evolved microbes from next-generation sequencing data using *breseq*. Methods Mol. Biol. 1151:165-188.

3. Winsor GL, Griffiths EJ, Lo R, Dhillon BK, Shay JA, Brinkman FS. 2016. Enhanced annotations and features for comparing thousands of *Pseudomonas* genomes in the *Pseudomonas* genome database. Nucleic Acids Res 44:D646-53

4. Kovach ME, Elzer PH, Steven Hill D, Robertson GT, Farris MA, Roop RM, Peterson KM. 1995. Four new derivatives of the broad-host-range cloning vector pBBR1MCS, carrying different antibiotic-resistance cassettes. Gene 166:175–6.

5. Smalley NE, Schaefer AL, Asfahl KL, Perez C, Greenberg EP, Dandekar AA. 2022. Evolution of the quorum sensing regulon in cooperating populations of *Pseudomonas aeruginosa*. MBio 13:e00161-22.

6. Kostylev, M, Smalley, NE, Chao, MH, & Greenberg, EP. 2023. Relationship of the transcription factor MexT to quorum sensing and virulence in *Pseudomonas aeruginosa*. J Bacteriol 205(12):e0022623.

7. Choi KH, Schweizer HP (2006) mini-Tn7 insertion in bacteria with single attTn7 sites: example *Pseudomonas aeruginosa*. Nat Protoc 1:153–161.

8. Zhao K, Tseng BS, Beckerman B, Jin F, Gibiansky ML, Harrison JJ, Luijten E, Parsek MR, Wong GCL. 2013. Psl trails guide exploration and microcolony formation in *Pseudomonas aeruginosa* biofilms. Nature 497:388–391.

9. Wang M, Schaefer AL, Dandekar AA, Greenberg EP. 2015. Quorum sensing and policing of *Pseudomonas aeruginosa* social cheaters. Proc Natl Acad Sci 112:2187–2191.

10. Feng X, Kostylev M, Dandekar AA, Greenberg EP. Dynamics of cheater invasion in a cooperating population of *Pseudomonas aeruginosa*. Sci Rep. 9(1):10190. doi: 10.1038/s41598-019-46651-5.

11. Kostylev M, Kim DY, Smalley NE, Salukhe I, Greenberg EP, Dandekar AA. Evolution of the *Pseudomonas aeruginosa* quorum-sensing hierarchy. Proc Natl Acad Sci U S A. 116(14):7027-7032. doi: 10.1073/pnas.1819796116.

12. Simon R, Priefer U, Pühler A. 1983. A broad host range mobilization system for in vivo genetic engineering: transposon mutagenesis in gram negative bacteria. Bio/Technology 1:784–791.
